# Ten-year follow-up results of a randomised controlled study comparing level-I *vs* level-III axillary lymph node dissection for primary breast cancer

**DOI:** 10.1038/sj.bjc.6603364

**Published:** 2006-10-03

**Authors:** H Kodama, Y Nio, C Iguchi, N Kan

**Affiliations:** 1 Kodama Breast Clinic, Kitano-kamihakubai-cho-35, Kita-ku, Kyoto, Japan

**Keywords:** breast cancer, axillary dissection, level-I dissection, level-III dissection, randomised control study

## Abstract

The most appropriate level of axillary dissection for breast cancer remains unclear. The present randomised study compared the treatment results of level-I *vs* level-III dissection in T1/2/3 and N0/1a/1b (1987 UICC classification) breast cancer without distant metastasis. Between 1995 and 1997, 522 patients were enrolled, and 514 were eligible. They were stratified into breast-conserving surgery or mastectomy, and then further stratified into level-III dissection (group-A, *n*=258) or level-I dissection (group-B, *n*=256). All patients were given oral 5-fluorouracil at 200 mg day^−1^ and tamoxifen at 20 mg day^−1^, daily for 2years. Group-A resulted in a significantly longer operation time (77.0 *vs* 60.5 min, *P*<0.0001) and significantly larger blood loss (62.1 *vs* 48.1 ml, *P*<0.0001) than group-B, but in no significant differences in the frequencies of arm oedema and shoulder disturbance. Group-A resulted in a significantly larger number of dissected nodes than group-B (18.7 *vs* 14.8, *P*<0.0001), but in no differences in the number of involved nodes (1.54 *vs* 1.44). There were no significant differences in the 10-year overall and disease-free survival rates: 89.6 and 76.6% for group-A *vs* 87.8 and 74.1% for group-B, respectively. In conclusion, level-III dissection resulted in a longer operation time and greater blood loss than level-I, but did not improve the survival rate. Level-III dissection is not a recommended surgery for T1–3/N0–1b breast cancer.

Lymph node (LN) metastasis and tumour size are significantly associated with the survival of patients with breast cancer. Previously, the extent, and recently, the number of involved LN nodes have become important prognostic factors that guide selection of those patients who might benefit from adjuvant treatment (Smith *et al*, 1977; Fisher *et al*, 1983). The regional LN of breast cancer includes axillary (Ax) and internal mammary nodes. Previously, an extended radical mastectomy, which removes internal mammary nodes as well as Ax nodes, was one of the standard surgeries for breast cancer; however, several randomised controlled studies demonstrated that internal mammary dissection was not useful to improve survival after surgery (Lacour *et al*, 1983; Meier *et al*, 1989; Veronesi *et al*, 1999). Accordingly, at present, most breast surgeons dissect only Ax nodes.

However, several studies indicated that Ax dissection did not help to improve the survival rate after surgery (Fisher *et al*, 1977; Fisher *et al*, 1985). Furthermore, a meta-analysis using very large samples also demonstrated that Ax dissection and radiotherapy did not improve survival after breast cancer surgery (Early Breast Cancer Trialists Collaborative Group, 1995), although several researchers still reported that Ax dissection produced a significantly higher survival rate than node-preserving surgery (Cabanes *et al*, 1992). Several clinical studies demonstrated that the incidence of Ax recurrence was very high (ranging between 18 and 35%), when clinically uninvolved Ax nodes were observed without Ax dissection or radiotherapy (Lythgoe and Palmer, 1982; Fisher *et al*, 1985; Ribeiro *et al*, 1993). Therefore, Ax dissection is the standard procedure for breast cancer surgery, with the aim of controlling local recurrence and classifying the tumour (pN) stage.

Ax dissection is classified into three levels: level-I (low-axilla), LNs lateral to the lateral border of pectoralis minor muscle; level-II (mid-axilla), LNs between the medial and lateral borders of pectoralis minor muscle and the interpectoral (Rotter) LNs; and level-III (apical axilla), apical LNs and those medial to the medial margin of the pectoralis minor muscle.

However, Ax dissection is associated with postsurgical complications, especially arm oedema and motion disturbance of the shoulder. Therefore, in order to reduce these complications by avoiding unnecessary Ax dissection, a sentinel node biopsy (SNB) or four-node biopsy has recently become popular. However, these procedures are indicated for N0 breast cancer. Furthermore, if SNB is positive, Ax LN should be dissected. At present, there is no consensus what level of Ax nodes should be dissected for N1 or node-positive breast cancer. Accordingly, the level of Ax dissection is not only an old but also a new issue in breast cancer surgery.

The NIH consensus conference recommended level-I or level-II dissection as standard surgery, and level-III dissection for patients with obviously involved LNs (NIH Consensus Conference, 1991). However, to our knowledge, there has been no randomised controlled study comparing level-I and level-III in terms of prognosis and complications. Therefore, at present, the most appropriate level of dissection remains still unclear, and the clinical significance of level-III dissection is one of the most pressing issues in breast cancer surgery.

The present randomised controlled study was designed and started in 1995 to compare the level-III dissection with the level-I dissection in breast cancer patients with T1/2/3 and N0/1a/1b, which were classified according to the UICC classification, 4th edition (UICC, 1987), with regard to overall survival, disease-free survival and QOL (quality of life) (especially arm oedema and motion dysfunction of the shoulder).

## PATIENTS AND METHODS

### Study design

Several basic criteria had to be met before patients were included in the study: (1) cytological proof of breast cancer, (2) curative surgery (mastectomy or breast-conserving surgery), (3) T1–3 (no direct invasion to the chest wall or skin), (4) N0 (no palpable Ax nodes), N1a (movable Ax nodes not considered to contain growth) or N1b (movable Ax nodes considered to contain growth) according to the 1987 UICC stage classification, 4th edition (UICC, 1987), (5) performance status <3 (ECOG scale) and (6) age under 81 years.

Contraindications to patient selection included: (1) a concomitant malignant disease, (2) prior surgery, chemotherapy, endocrine therapy, radiotherapy or immunotherapy for breast cancer and (3) an active infectious disease.

This clinical study was carried out by a single institute (Kodama Breast Clinic, Kyoto, Japan). The end point was disease-free or overall survival after surgery, and the trial was originally designed to detect a difference in the 5- and 10-year survival rates between the level-III dissection group (group-A) and the level-I dissection group (group-B). Several studies reported that about one-third (22–45%) of clinically node-negative breast cancers had LN metastases (Sacre, 1983; Noguchi *et al*, 1993; Yang *et al*, 1996), and about one third (22–43%) of patients with level-I or level-II metastases had level-III metastases (Rosen *et al*, 1983: Veronesi *et al*, 1987). The previous statistics demonstrated that about 30% of patients with T1–3/N0–1 breast cancer in our clinic had nodal involvement, and about 10% had level-III metastases. Accordingly, if level-III metastases were left without dissection, these metastases may have affected the prognosis of the patients, and it was expected that there would be about 10% difference in the disease-free survival rate and about 5% in the overall survival rate between the level-I and level-III dissection groups. Based on this hypothesis, when the expected difference in survival rates between the groups was estimated as more than 5 (85 *vs* 90%), with 0.05 for alpha-error and 0.05 for beta-error, during the 13 years of the study (3 years for recruiting and an additional 10 years for follow-up), the sample size (number of patients) needed in each group was calculated to be 277 for each arm (totally 554) (Schoenfeld and Richter, 1982). Based on the above, the study target was to accumulate at least 275 patients for each arm (a total of 550).

### Informed consent and the ethics committee

All patients and their families were fully informed with regard to the study aim, treatment programme, and expected results and clinical benefits, such as overall survival, disease-free survival and QOL, and informed consent was then obtained. The study and protocol were supervised and reviewed by the extramural the Ethics and Safety Committee (Professor Syunzo Maetani, Kyoto University and Dr Kazuhisa Ohgaki, Kyoto Police Hospital).

### Patient registration and randomisation

The study was open to patients from January 1995. After informed consent was obtained, prerandomisation was stratified with regard to the method of surgery, breast-conserving surgery/mastectomy and then the patients were pre-enrolled into the registration centre by fax one day before surgery. According to Zelen's design (Zelen, 1979), the patients were prerandomised with minimisation to balance the prognostic factors in individual institutes, with regard to T – primary tumour (T0/1/2/3), N – nodal involvement (N0/1a/1b) and menopause (pre/post: if the menopausal state was unknown, patients over 50 were classified as menopause).

**Figure 1:**
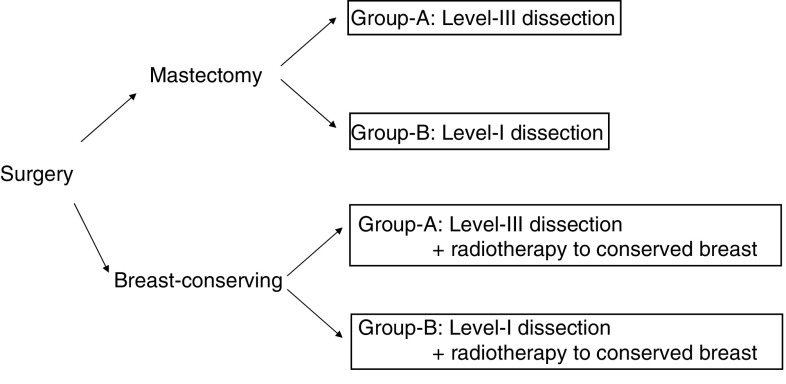
Stratification of the patients. From 1week after surgery, all patients received oral 5-FU at 200 mg day^−1^ and tamoxifen at 20 mg day^−1^ for 2 years.

On the day of the surgery, according to the fax on direction of the randomisation from the registration centre, the patients who received each surgery were randomly assigned to one of two groups, group-A and group-B, and underwent an assigned level of Ax dissection (Figure 1). After surgery, the stage of breast cancer was classified according to the UICC (TNM) stage classification system, 1987, 4th edition (UICC, 1987). The registration centre was located at the Department of Medical Technology, Kyoto University School of Medicine, and the randomisation was directed by Professor Syunzo Maetani and his staffs.

### The treatment protocol

The treatment protocol is summarised in Figure 1. The patients were first assigned into the two arms: mastectomy and breast-conserving surgery groups, and then each group were further classified into two arms, group-A (level-III dissection) and group-B (level-I dissection), respectively. From the seventh day after surgery, all patients were orally administered 5-fluorouracil (5-FU) at 200 mg body^−1^ day^−1^ and tamoxifen (TAM) at 20 mg body^−1^, daily for 2 years. The patients, who underwent breast-conserving surgery, received radiotherapy to the remnant breast at 2.0 Gy × 25 times (total dose of 50 Gy) from the 14th day after surgery.

Examinations of haematology, serum biochemistry, serum tumour markers and evaluation of symptomatic and performance status were routinely performed. If recurrence appeared, the patients were offered alternative regimens.

### Evaluation of postsurgical complications

The present study was also designed to compare the frequency of postsurgical complications, such as an arm oedema (swelling) and Ax fluid retention, which are characteristic of breast cancer surgery, in level-III *vs* level-I dissection. Arm oedema (swelling) was evaluated according to the maximum circumference size of the operated-side arm in comparison with the contralateral arm as follows: (1) severe, 3 cm or larger; (2) moderate, larger within 1–3 cm; and (3) negative, same or less than 1 cm larger. The motion dysfunction of the shoulder was evaluated as follows: (1) severe, disturbance in daily life or frozen shoulder; (2) moderate, muscle atrophy or weakness without disturbance in daily life; and (3) negative, no disturbance.

The lymph oedema and the arm dysfunction were evaluated by two staffs, the doctor and the nurse, separately, who were blinded to the extent of surgery, routinely at 3-month intervals for 2 years after surgery.

### Patient follow-up

All patients were followed by physical examination, general X-ray examination, ultrasonography, computed tomography, routine haematologic and biochemical examinations, and serum tumour marker assays. The postsurgical status of all patients was surveyed on December 2005. The median follow-up period was 9.3 years.

### Statistical analysis

During the course of this 10-year study, the UICC stage classification changed from the 4th edition to the 6th edition (UICC, 2002). Therefore, the final results were analysed according to the new classification. Chi-squared and Mann–Whitney *U*-tests were used to compare the backgrounds of patients between each group. The overall survival and disease-free survival were the true end points. Survival was calculated by the Kaplan–Meier method. A statistical comparison of the survival rates among the three groups was made by the generalised-Wilcoxon test. Multivariate analysis of the maximum-likelihood estimates using Cox's proportional-hazard model was used to obtain the conditional risk of breast cancer-related death. All analyses were performed using StatView software (SAS Institute Inc., Cary, NC, USA), and a *P*-value less than 0.05 was considered statistically significant.

## RESULTS

A total of 522 patients were registered, and eight patients were ineligible: seven for distant metastasis to bone and one for level-II dissection. As a result, a total of 514 patients were eligible for analysis in the study. The comparisons among background factors of the eligible patients are summarised in Table 1. Group-A included 96 mastectomies and 162 breast-conserving surgeries, and group-B included 95 mastectomies and 161 breast-conserving surgeries. There were no significant differences in the background factors, such as clinical stage, histology and age.

The surgical procedures and the related complications were summarised in Table 2. The level-III dissection usually took longer time and more blood loss for surgery than the level-I dissection: the mean operation time was 77.0 min for group-A *vs* 60.5 min for group-B (*P*<0.0001) and the mean bleeding volume was 62.1 ml for group-B *vs* 48.1 ml for group-A (*P*<0.0001). There were no significant differences in the frequencies of postsurgical arm oedema and shoulder disturbance between group-B and group-A.

The distribution of dissected nodes was summarised in Table 3. The mean number of dissected nodes was 18.7 for group-A *vs* 14.8 for group-B, and the level-III dissection resulted in significantly larger number of dissected nodes (*P*<0.0001). However, there were no differences in the number of involved nodes between group-A (1.54) and group-B (1.44). Table 4 summarised a distribution of involved nodes at each dissection levels in group-A. Out of 88 patients with nodal metastases, 61 (23.6%) had metastases only at level-I nodes. The metastases beyond the level-II were seen in 27 patients (10.5%): level-I and -III, 14 (5.4%); level-I and -II, 4 (1.6%); level-I, -II and -III, 4 (1.6%); level-II alone, 4 (1.6%); and level-III alone, only one patient (0.4%).

The recurrences were summarised in Table 5. The recurrences were seen in 53 (20.6%) patients in group-A and 58 (22.7%) patients in group-B. The first recurrence was seen most frequently at LNs in both groups: 18 in group-A and 16 in group-B, and most of them were seen at supraclavicular nodes: 15 in group-A and 15 in group-B, and at the Ax nodes no recurrence was seen in group-A and the only one recurrence was seen in group-B. Local recurrences were seen in 15 patients in group-A and 13 in group-B. Distant metastases were seen in 20 patients in group-A and 28 in group-B, and bone and lung were the major sites of the first recurrence. There were no significant differences in the frequency or site of the first recurrence.

**Figure 2:**
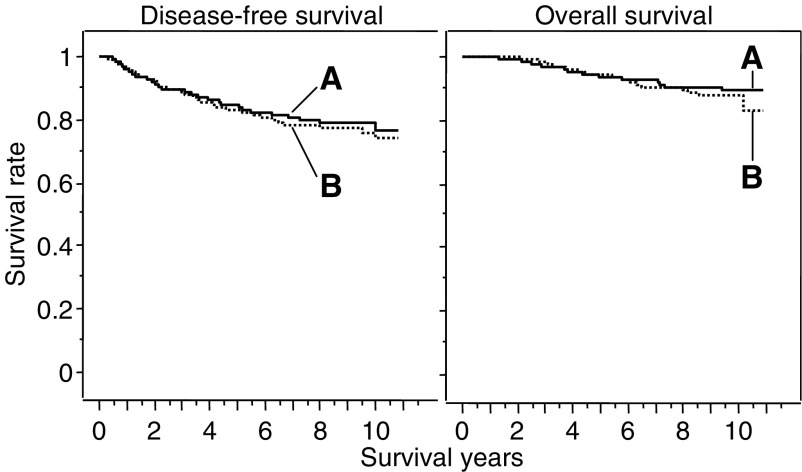
Overall survival curves and disease-free survival curves. The 5- and 10-year overall survival rates were 93.6 and 89.6% for group-A (*n*=258) *vs* 94.5 and 87.8% for group-B (*n*=256), respectively (*P*=0.5526). The 5- and 10-year disease-free survival rates were 84.7 and 76.6% for group-A *vs* 83.1 and 74.1% for group-B, respectively (*P*=0.6137).

The survival rates are summarised in Table 6 and Figure 2. There were no significant differences in the overall survival and disease-free survival between group-A and group-B (Figure 2). The 5- and 10-year overall survival rates were 93.6 and 89.6% for group-A *vs* 94.5 and 87.8% for group-B, respectively (*P*=0.5526). The 5- and 10-year disease-free survival rates were 84.7 and 76.6% for group-A *vs* 83.1 and 74.1% for group-B, respectively (*P*=0.6137).

Multivariate analyses indicated that pT, pN and oestrogen receptor expression were significant variables for overall survival, and age, pT and pN were significant variables for disease-free survival (Table 7). The level of Ax dissection was not a significant variable for either overall survival or disease-free survival.

## DISCUSSION

In the present study, LN metastases were seen in 30.1–32.2% of the patients at level-I LNs, in 4.7% at level-II LNs and 7.4% at level-III LNs. Of these, level-I metastases alone were seen in 23.6%, and level-II and/or level-III metastases were seen in 10.5% of the patients, suggesting that level-I dissection alone results in an increase in the recurrence rate and a decrease in survival rates, equivalent to about 10% (27 patients). The present study aimed to clarify this issue, and the results indicated that there were no differences in overall survival and disease-free survival between the level-I and level-III dissection groups in patients with N0 or N1a breast cancer. Furthermore, there were no differences in arm oedema or motion disturbance of the shoulder between level-I and level-III dissection groups. The advantage of level-I dissection was seen in the surgical procedure, and the operation time and blood loss were significantly shorter and smaller than those for level-III dissection. These results were expected, considering the complicated procedure for level-III dissection.

The overall recurrence rates were 22.7% for level-I and 20.6% for level-III, and there were no significant differences between them. With respect to Ax recurrence, only one Ax recurrence was seen in the level-I group and no Ax recurrence was seen in the level-III group. Most LN recurrences were seen at the supraclavicular LNs, and there were no differences in its incidence between the level-I and level-III groups. Furthermore, there were also no differences in local recurrence between the two groups. Accordingly, the present results indicated that level-III dissection did not improve the local control in comparison with level-I dissection.

Distant metastases were seen in 28 patients (10.9%) of group-B (level-I) and in 20 patients (7.8%) of group-A (level-III), and the initial sites of the distant metastases were seen in bone or the lungs in most cases in both groups. There were no significant differences between them. Therefore, level-III dissection did not contribute to inhibiting distant metastases.

The above results suggest that level-I dissection may be adequate for breast cancer of T1–3 with N0–1b stage. In the present study, the patients in these categories were expected to have a 10.5% rate (about 27 cases) of metastases in level-II and level-III LNs. Accordingly, level-III dissection was expected to improve survival or recurrence rates, but level-III dissection did not improve the survival rate or recurrence rate equivalent to this value. In addition, multivariate analyses indicated that the level of Ax dissection was not a significant variable for overall survival and disease-free survival, and these results also support the idea that the level of Ax dissection has no influence on the prognosis after breast cancer surgery.

It is unclear why there were no differences in overall survival, disease-free survival and the recurrence rate between level-I and level-III dissections. The present results demonstrated that level-III dissection removed only four more LNs than level-I dissection, and four LNs may be too small to produce a significant difference in the patients' outcome, and this may be one of the reasons. However, recurrence was not seen at level-II or level-III areas in group-B, suggesting that these LN metastases might have spontaneously disappeared in group-B patients. These results are compatible with previous studies on internal mammary dissection. Metastases to internal mammary nodes were seen in about 20% of breast cancer cases (Lacour *et al*, 1976), and 9.1–13.8% of patients without Ax node metastases had internal mammary nodes metastases (Urban and Castro, 1971; Veronesi *et al*, 1985). Previously, in order to remove internal mammary nodes, a variety of extended radical mastectomy techniques were applied in breast cancer surgery; however, several randomised controlled studies demonstrated that internal mammary dissection did not improve the survival after surgery and the frequency of recurrence at the internal mammary nodes was lower than expected (Lacour *et al*, 1983; Meier *et al*, 1989; Veronesi *et al*, 1999).

The reasons why involved nodes disappeared are unclear, but we have three possible hypotheses: one is that the postsurgical adjuvant therapies were effective at controlling the local growth of the tumours. In the present study, the patients received an adjuvant therapy with oral 5-FU and TAM. TAM is the gold standard for adjuvant endocrine therapy after breast cancer surgery (Early Breast Cancer Trialists' Collaborative Group, 1998; Stewart *et al*, 2001). However, oral 5-FU as adjuvant therapy is not popular in the USA and Europe. In Japan, oral chemotherapy with 5-FU or its derivatives has been widely applied as a beneficial adjuvant therapy after breast cancer surgery (Tominaga *et al*, 2003; Noguchi *et al*, 2005; Shimane Breast Cancer Study Group, 2006), and this study may also support the survival benefits of oral adjuvant chemotherapy with 5-FU or its derivatives. The second possibility is that microscopic residual tumour cells might be eliminated by an immunosurveillance system, which may be activated by removal of the main tumours. The third possibility is that the nodes located at the central site of the dissected nodes may be withdrawn due to the shut down of lymphatic circulation, even if they are involved.

The present results indicate that level-III dissection is not necessary for N0–1b (N0–1 by 2002 UICC) breast cancer, and suggest that if SNB is positive, the level-I dissection is indicated. This is a very important indication for breast-conserving surgery. SNB is indicated for N0 breast cancers, and most of them are also indicated for breast-conserving surgery. Since level-III LNs locate behind the major pectoral muscle, the procedure of level-III dissection is not so easy for breast-conserving surgery with a small Ax incision for SNB and sometimes an additional large incision, which may result in a cosmetic problem, if necessary.

As discussed above, the present study indicated that level-III dissection is not necessary for T1–3/N0–1b breast cancer.

## CONCLUSION

The present study indicated that level-III dissection did not improve the survival rate, but did result in a longer operation time and a greater blood loss than level-I dissection. Level-III dissection is not a recommended surgery for T1–3/N0–1b breast cancer.

## Figures and Tables

**Table 1:** Background of the eligible patients

	**Group-A (level-III), *n*=258**	**Group-B (level-I), *n*=256**	**Statistics**
Age: mean±s.d.	50.6±10.1	51.6±10.6	NS
Menopause (or >50 years)	127	131	NS
Premenopause (⩽50 years)	131	125	
			
*ER*			
(+)	161	141	
(−)	75	95	
Unknown	22	20	
			
*T-factor*			
Preoperative evaluation			
T1	113	118	NS
T2	139	130	
T3	6	8	
Postsurgical evaluation			
Tis	12	7	NS
pT1	108	114	
pT2	131	127	
pT3	7	8	
			
*N-factor*			
Preoperative evaluation (1987 UICC, 4th edition)			
N0 (no palpable nodes)	152	149	NS
N1a (movable but not considered no growth)	74	66	
N1b (movable considered to contain growth)	32	41	
Postsurgical evaluation (2002 UICC, 6th edition)			
pN0	170	179	NS
pN1 (1–3 positive nodes)	62	49	
pN2 (4–9 positive nodes)	10	19	
pN3 (10 or more positive nodes)	16	9	
Preoperative stage (1987 UICC, 4th edition)			
I	183	195	NS
II	54	60	
III	21	1	
Postsurgical stage (2002 UICC, 6th edition)			
0	12	7	NS
I	77	92	
II	143	128	
III	26	29	
			
*Surgery*			
Mastectomy	96	95	NS
Breast-conserving surgery	162	161	

ER=oestrogen receptor; NS=no significant differences; UICC=International Union Against Cancer.

**Table 2:** Surgery and its related complications

	**Group-A (level-III)**	**Group-B (level-I)**	**Statistics**
Operation time (min)	77.0±14.2	60.5±9.8	*P*<0.0001
Bleeding (ml)	62.1±39.6	48.1±29.2	*P*<0.0001
Number of removed LNs	18.7±5.5	14.8±4.6	*P*<0.0001
			
Postsurgical complication			
Arm oedema	15 (5.8%)	14 (5.5%)	NS
Shoulder disturbance	22 (8.5%)	21 (8.2%)	NS

LN=lymph node; NS, no significant differences.

Values indicate mean±s.d.

**Table 3:** Results of the Ax dissection

	**Group-A (level-III), *n*=258**	**Group-B (level-I), *n*=256**	**Statistics**
*Level-I*			
Involved nodes	1.29±3.25	1.44±4.15	NS
Dissected nodes	13.73±4.12	14.81±4.65	*P*=0.0057
			
*Level-II*			
Involved nodes	0.07±0.35		
Dissected nodes	1.08±1.39		
			
*Level-III*			
Involved nodes	0.18±0.73		
Dissected nodes	3.89±1.98		
			
*Total*			
Involved nodes	1.54±3.92	1.44±4.15	NS
Dissected nodes	18.72±5.47	14.81±4.65	*P*<0.0001

Ax=axillary; NS=no significant difference.

**Table 4:** Distribution of involved nodes in level-III dissection

	**Involvement**	**Group-A (level-III dissection), *n*=258**	**Group-B (level-I dissection), *n*=256**
*Level*			
I	(−)	175 (67.8%)	179 (69.9%)
	(+)	83 (32.2%)	77 (30.1%)
II	(−)	246 (95.3%)	
	(+)	12 (4.7%)	
III	(−)	239 (92.6%)	
	(+)	19 (7.4%)	
			
*Combinations*			
I(−)/II(−)/III(−)		170 (65.9%)	
I(+)/II(−)/III(−)		61 (23.6%)	
I(+)/II(+)/III(−)		4 (1.6%)	
I(+)/II(−)/III(+)		14 (5.4%)	
I(+)/II(+)/III(+)		4 (1.6%)	
I(−)/II(+)/III(−)		4 (1.6%)	
I(−)/II(−)/III(+)		1 (0.4%)	

**Table 5:** Postsurgical recurrence

	**Group-A (level-III), *n*=258**	**Group-B (level-I), *n*=256**	**Statistics**
Recurrence	53 (20.55%)	58 (22.65%)	NS
			
*Primary recurrent site*			
Local recurrence	15 (5.81)	13 (5.08%)	
Contralateral breast	0 (0%)	1 (0.39%)	
Nodal metastasis	18 (6.98%)	16 (6.25%)	
Axillary	0 (0%)	1 (0.39%)	NS
Contralateral axillary	1 (0.39%)	0 (0%)	
Supraclavicular	15 (5.81%)	15 (5.86%)	
Parasternal	2 (0.78%)	0 (0%)	
Distant metastasis	20 (7.8%)	28 (10.9%)	NS
Bone	8 (3.10%)	11 (4.30%)	
Lung	7 (2.71%)	10 (3.91%)	
Liver	3 (1.16%)	5 (1.95%)	
Others	2 (0.78%)	2 (0.78%)	
			
*Death due to*			
Breast cancer	24 (9.30%)	29 (11.33%)	NS
Other diseases	6 (2.33%)	6 (2.35%)	

NS=no significant difference.

**Table 6:** Postsurgical survival rates

	**Group-A (level-III) (%)**	**Group-B (level-I) (%)**	**Statistics**
*I. Overall survival*			
5-year	93.6	94.5	
10-year	89.6	87.8	NS
			
*II. Disease-free survival*			
5-year	84.7	83.1	
10-year	76.6	74.1	NS

NS=no significant difference.

**Table 7:** Multivariate analyses using Cox's proportional-hazard risk model

	**Overall survival**		**Disease-free survival**	
**Variable**	**Risk ratio (95% confidence)**	***P*-value**	**Risk ratio (95% confidence)**	***P*-value**
Age	0.988 (0.960–1.017)	0.4143	0.964 (0.945–0.983)	0.0003
pT (1/2/3)	2.293 (1.353–3.884)	0.0020	2.254 (1.563–3.250)	<0.0001
pN (0/1/2/3)	2.073 (1.568–2.740	<0.0001	2.191 (1.804–2.662)	<0.0001
ER (0/1)	0.395 (0.222–0.701)	0.0015	0.848 (0.571–1.259)	0.4136
Level of dissection (1/3)	0.964 (0.721–1.290)	0.8067	0.995 (0.8151–1.215)	0.9634
No. of dissected nodes	0.987 (0.938–1.039)	0.6156	0.969 (0.934–1.006)	0.1032

ER=oestrogen receptor.
